# Genomic erosion and extensive horizontal gene transfer in gut-associated Acetobacteraceae

**DOI:** 10.1186/s12864-019-5844-5

**Published:** 2019-06-10

**Authors:** Bryan P. Brown, Jennifer J. Wernegreen

**Affiliations:** 1 Nicholas School of the Environment, Duke University, 9 Circuit Dr., Durham, NC 27710 USA; 2 Genomic and Computational Biology, Duke University, 101 Science Dr., Durham, NC 27705 USA; 3 Center for Global Infectious Disease Research, Seattle Children’s Research Institute, 1900 9 Ave., Seattle, WA 98101 USA

**Keywords:** Gut microbiota, Evolution, Insects, HGT, AAB, Genomic erosion

## Abstract

**Background:**

Symbiotic relationships between animals and bacteria have profound impacts on the evolutionary trajectories of each partner. Animals and gut bacteria engage in a variety of relationships, occasionally persisting over evolutionary timescales. Ants are a diverse group of animals that engage in many types of associations with taxonomically distinct groups of bacterial associates. Here, we bring into culture and characterize two closely-related strains of gut associated Acetobacteraceae (AAB) of the red carpenter ant, *Camponotus chromaiodes*.

**Results:**

Genome sequencing, assembly, and annotation of both strains delineate stark patterns of genomic erosion and sequence divergence in gut associated AAB. We found widespread horizontal gene transfer (HGT) in these bacterial associates and report elevated gene acquisition associated with energy production and conversion, amino acid and coenzyme transport and metabolism, defense mechanisms, and lysine export. Both strains have acquired the complete NADH-quinone oxidoreductase complex, plausibly from an Enterobacteriaceae origin, likely facilitating energy production under diverse conditions. Conservation of several lysine biosynthetic and salvage pathways and accumulation of lysine export genes via HGT implicate L-lysine supplementation by both strains as a potential functional benefit for the host. These trends are contrasted by genome-wide erosion of several amino acid biosynthetic pathways and pathways in central metabolism. We perform phylogenomic analyses on both strains as well as several free living and host associated AAB. Based on their monophyly and deep divergence from other AAB, these *C. chromaiodes* gut associates may represent a novel genus. Together, our results demonstrate how extensive horizontal transfer between gut associates along with genome-wide deletions leads to mosaic metabolic pathways*.* More broadly, these patterns demonstrate that HGT and genomic erosion shape metabolic capabilities of persistent gut associates and influence their genomic evolution.

**Conclusions:**

Using comparative genomics, our study reveals substantial changes in genomic content in persistent associates of the insect gastrointestinal tract and provides evidence for the evolutionary pressures inherent to this environment. We describe patterns of genomic erosion and horizontal acquisition that result in mosaic metabolic pathways. Accordingly, the phylogenetic position of both strains of these associates form a divergent, monophyletic clade sister to gut associates of honey bees and more distantly to *Gluconobacter.*

**Electronic supplementary material:**

The online version of this article (10.1186/s12864-019-5844-5) contains supplementary material, which is available to authorized users.

## Background

Symbiotic associations between animals and microbial partners are dynamic and influence genomic evolution in myriad ways. Persistent relationships between animals and microbiota have facilitated a range of adaptations, from niche diversification [1, 2] to dramatic increases in metabolic potential [3], and, in select cases, even leading to speciation of one or multiple partners [4]. Studies of animal-bacterial coevolution have explored several ancient endosymbioses [5], in which millions of years of shared evolutionary history have profoundly impacted bacterial genome evolution [5, 6]. In addition, more recent associations, including with gut microbiota, also represent an attractive system for studying the genomic consequences of symbiosis, due to their persistence across a diverse range of hosts species, and their variable level of intimacy with a given host [7].

Comparative analyses of gut associates have revealed varied levels of host specialization and diverse genomic outcomes. For example, extracellular gut associates of pentatomid bugs display remarkable genome erosion, AT-biased nucleotide composition, accelerated rates of molecular evolution, and strict co-cladogenesis with the host [8]. Gut associates of social bees possess specialized genomes adapted to their specific host species [7] and contribute to pectin degradation in the gastrointestinal tract [9]. Likewise, in vertebrate hosts, persistent gut symbionts show patterns of host specialization evident by monophyletic clades associated with distinct species [10], though their genomes have retained moderate base compositions and show considerable strain-level variation in gene content between host populations [11].

Among animals, the diverse family Formicidae (the Ants) offers excellent models to explore host-symbiont coevolution. Bacterial communities associated with ant guts vary widely across host families but tend to show a relatively high level of stability within a host species [12–15]. Camponotine ants enjoy a nearly worldwide range and, despite this vast distribution, regularly associate with select taxa in the Acetobacteraceae (AAB) [12, 13, 16] and Lactobacillaceae (LAB) [17–19]. Previous work has suggested that camponotine ants may acquire secondary symbionts from other insect hosts through their diet, providing an opportunity for co-option of persistent gut associates [20]. Based on 16S rDNA amplicon sequencing, we have found that two lineages within the Acetobacteraceae, AAB1 and AAB2, occur in greater than 50% of host colonies (unpublished data). Though the origin of these associates remains unclear, they are genetically distinct from all environmental or other insect-associated Acetobacteraceae.

In an evolutionary analysis of 16S rDNA, we previously showed that gut associated Acetobacteraceae (strains AAB1 and AAB2) [12] display elevated mutation rates, AT bias, significant destabilization of 16S rRNA, and monophyly with strains only found in other ants. Deep divergence from environmental isolates and host-restricted distribution suggest that AAB1 and AAB2 may share a long history with their host group. Therefore, *Camponotus* AAB represent an intriguing system for assessing the evolutionary processes facilitating host-symbiont interactions and adaptation.

Here, we culture representatives of one lineage (AAB2) associated with the red carpenter ant, *Camponotus chromaiodes,* and perform whole genome sequencing, generating fully closed genomes of two strains of AAB2*.* Our analyses delineate various facets of genomic evolution in these gut associates, namely genomic erosion and extensive horizontal gene transfer, and we discuss how these changes may facilitate adaptation to the host gut. Our results are the first to describe the genomes of these symbiotic Acetobacteraceae and illustrate evolution in these gut associated bacteria.

## Results

### Genome characteristics

**Fig. 1:**
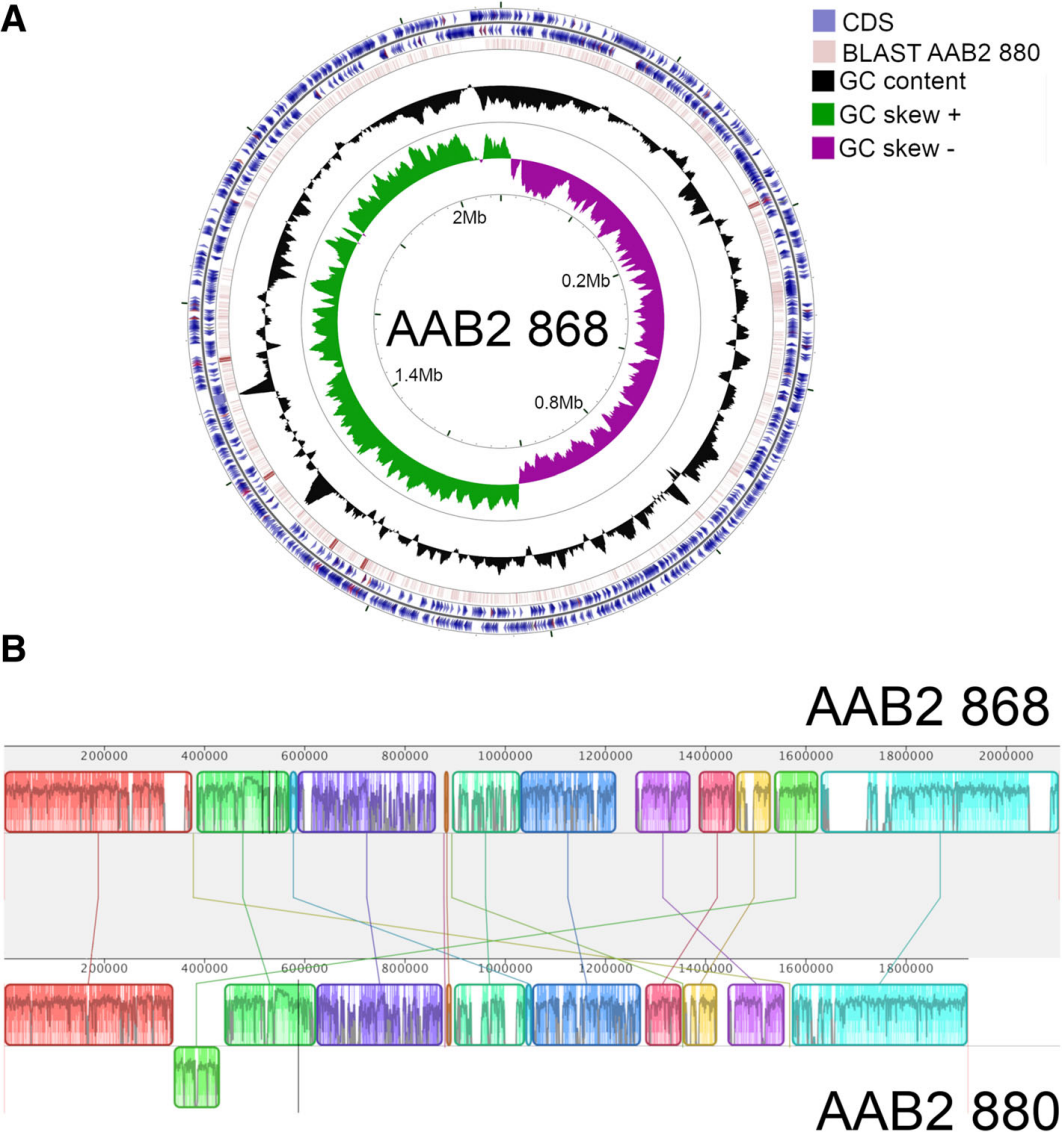
Chromosome maps and whole genome alignment of both AAB strains sequenced. **a**. Genome structure of AAB2 strain 868. From outside to the center, rings represent genes on forward strand, genes on reverse strand (tRNAs and rRNAs red), BLASTx alignment of AAB2 strain 880 against AAB2 868, GC content, and GC skew. **b**. Whole genome alignment of strains 868 (reference) and 880. Alignments are colored by locally collinear blocks

Isolates for full genome sequencing were obtained from pure cultures. We sequenced two isolates of the previously described acetic acid bacterial lineage, AAB2. Sequencing on the PacBio RSII generated datasets with mean read counts of 76,659 and mean read N50 of 23,501 bp. When assembled, the read datasets formed closed genomes with mean coverage of 443-fold and consensus concordance of 99.9967%. The AAB2 strain 868 genome is a circular, 2.1 Mb chromosome encoding 2032 CDS, including 342 putatively horizontally transferred genes (Table 1; Fig. 1). Strain 868 also possesses a 21.47 kb plasmid that encodes 32 genes. AAB2 strain 880 is a circular chromosome of 1.9 Mb encoding 1798 CDS, including 276 putatively horizontally transferred genes (Table 1; Fig. 1).

**Table 1:** Genome characteristics of sequenced strains and related isolates

Genome Name/Sample Name	IMG Genome ID	Genome Size	Gene Count	CRISPR Count	GC %	CDS Count	CDS %	rRNA Count	Pseudogene Count	Horizontally Transferred Count	Horizontally Transferred %	Lifestyle
Acetobacteraceae bacterium 868	**2734482192**	**2107276**	**2111**	1	**43**	**2032**	**96.26**	**15**	14	**342**	**16.2**	**Host associated**
Acetobacteraceae bacterium 880	**2716885010**	**1923586**	**1874**	2	**43**	**1798**	**95.94**	**15**	7	**276**	**14.73**	**Host associated**
Roseomonas rosea DSM 14916	2585427600	5342582	5093	0	71	5027	98.7	10	NA	60	1.18	Free-living
Saccharibacter sp. AM169	2617271045	1978091	1785	2	59	1723	96.53	6	NA	37	2.07	Host associated
Gluconobacter oxydans 621H	637000122	2922384	2742	0	61	2664	97.16	12	NA	3	0.11	Free-living
Acetobacter aceti ATCC 23746	2516653054	3690768	3510	3	57	3442	98.06	9	130	195	5.56	Free-living
Acetobacter tropicalis DmCS_006	2609460235	3746694	3494	2	55	3436	98.34	3	NA	81	2.32	Host associated
Gluconobacter japonicus R-49117	2684622537	3151336	3071	0	56	3015	98.18	3	NA	56	1.82	Free-living
Acetobacter tropicalis NBRC 16470	2667527850	3500391	3248	3	56	3190	98.21	3	NA	75	2.31	Free-living
Acetobacter pomorum DmCS_004	2617271320	2843884	2750	2	52	2693	97.93	3	NA	19	0.69	Host associated
Acetobacter malorum LMG 1552	2713897234	344275	3250	0	58	3194	98.28	3	NA	62	1.91	Free-living
Azospirillum brasilense FP2	2551306490	6885108	6972	3	68	6889	98.81	2	NA	9	0.13	Free-living
Escherichia coli 100269_aEPEC	2698536335	5098872	5163	0	50	4948	95.84	10	NA	3	0.06	Free-living
Asaia platycodi SF2.1	2574180177	3528906	3054	NA	60	3005	98.4	49	44	184	5.89	Host associated
Acetobacter malorum DmCS_005	2597490292	3512607	3125	5	57	3066	98.11	4	NA	3	0.10	Host associated

Entries in bold indicate bacterial strains whose genomes were fully sequened in this study

Generally, all assemblies had high coverage and accuracy. The genome of AAB2 strain 868 assembled with 386.4-fold coverage spanning 2,107,145 bps with 99.9941% consensus concordance. The plasmid assembly had 99-fold coverage and 99.873% consensus concordance (though we recognize that is also possible that plasmid copy number declined during culturing). We also appreciate that PacBio large insert library construction is biased against shorter molecules and may have preferentially sequenced larger genomic fragments. We further aligned all quality-filtered Illumina reads to the complete plasmid assembly, finding a similar depth of coverage as that of the genome (Additional file 3: Figure S3, Additional file 4: Figure S4 and Additional file 5: Figure S5). The genome of AAB2 strain 880 assembled with 570.93-fold coverage spanning 1,923,512 bps with 99.9969% consensus concordance.

Compared to free living and other host associated isolates within the Acetobacteraceae, both strains of AAB2 displayed distinct differences. On average, the genomes from both strains were 1.2 Mb smaller than other acetic acid bacteria and had a mean GC content that was approximately 15% lower (Table 1). Additionally, both AAB2 strains possessed an average of 1221 fewer CDS than other Acetobacteraceae. We detected 14 pseudogenes in strain 868 and 7 pseudogenes in strain 880, as detected by loss of function mutations. These mutations resulted from frameshifting indel mutations and/or the presence of internal stop codons. With the exception of a putative amino acid permease in strain 868, all pseudogenes were annotated as hypothetical proteins.

### Metabolic pathway reconstruction

**Fig. 2:**
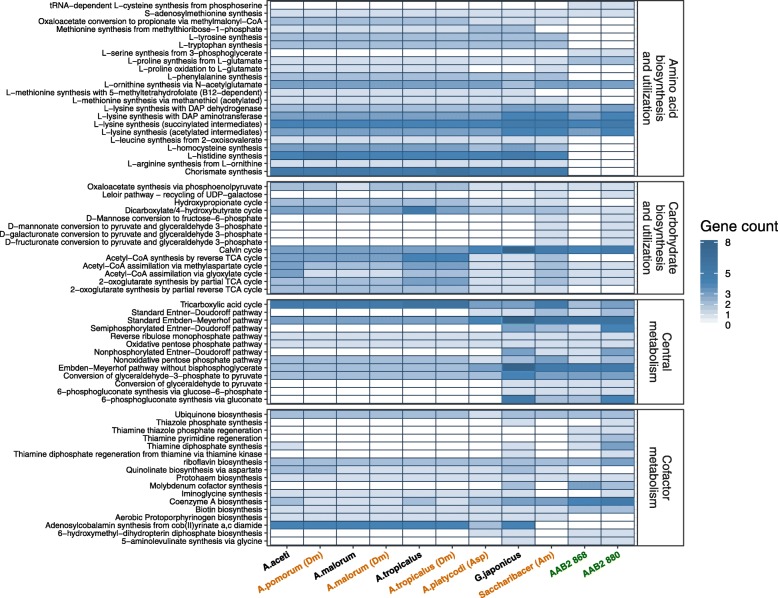
Abundance profiles of functions annotated by IMG networks. Distribution of genes affiliated with various IPWAY pathways (IMG Pathway). Pathways are binned according to the broader functional network in which they are affiliated. Labels of ant associates fully sequenced in this study are colored green, other host associated bacterial isolates are colored orange, and environmental isolates are colored black. Hosts are indicated in parentheses, where applicable (Dm: *Drosophila melanogaster; Asp: Anopheles* species; Am: *Apis mellifera*)

**Fig. 3:**
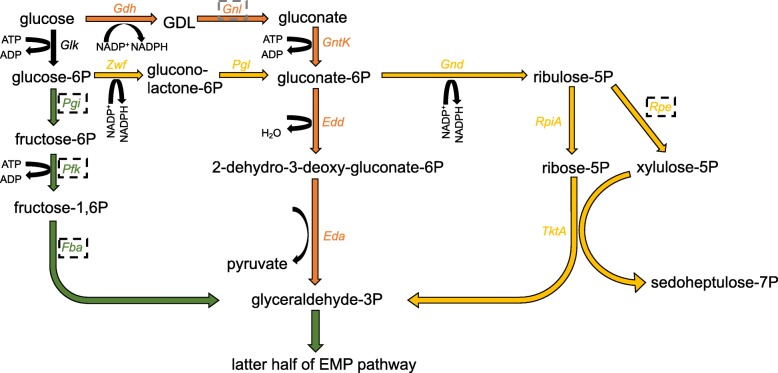
The mosaic glycolytic route in AAB2 strains 868 and 880. Key reactions in the standard EMP pathway of glycolysis are shown in green; the OPP pathway is displayed in yellow, and the ED pathway is shown in orange. Enzymes and reactions shown in black are shared by multiple pathways. Enzymes missing in both strains are outlined with a dashed black line, and enzymes missing from one strain are outlined with a dashed grey line. Gdh, glucose dehydrogenase; GDL, glucono delta-lactone; Gnl, gluconolactonase; Glk, glucokinase; GntK, gluconokinase; Zwf, glucose-6-phosphate dehydrogenase; Pgl, 6-phosphogluconolactonase; Gnd, 6-phosphogluconate dehydrogenase; Pgi, phosphoglucose isomerase; Edd, phosphogluconate dehydratase; RpiA, ribose-5-phosphate isomerase A; Rpe, ribulose-5-phosphate; Pfk, 6-phosphofructokinase; Fba, fructose bisphosphate aldolase; Eda, KDPG aldolase; TktA, transketolase 1

Relative to free living acetic acid bacteria (Additional file 1: Figure S1), the size and metabolic potential of genomes of both strains of AAB2 are shifted, mainly through gene loss. This trend of genome reduction is consistent among specialized gut associates of insects [7, 21]. Both strains have shared gene loss in several amino acid biosynthetic pathways as well as multiple central metabolic pathways such as glycolysis, oxidative pentose phosphate, and the tricarboxylic acid cycle (TCA; Fig. 2). Biosynthetic pathways that have undergone gene loss and are putatively nonfunctional include synthetic pathways for several amino acids and precursors (Fig. 2). The synthetic pathway for chorismate, a precursor molecule to the three aromatic amino acids, tyrosine, tryptophan, and phenylalanine, is entirely absent, as are synthetic pathways for each amino acid (Fig. 2). Furthermore, both strains appear to have lost the ability to fully or partially synthesize several other amino acids, though appear to have acquired select biosynthetic pathways for valine and serine, likely from horizontal transfer (Fig. 2). Additionally, both strains have retained serine-glycine interconversion enzymes and the ability to synthesize serine from phosphoserine and 5,10-Methylenetetrahydrofolate. However, the ability to synthesize serine from pyruvate and interchange with other amino acids is absent or reduced compared to other AAB. With respect to carbohydrate utilization and metabolism, both strains have undergone significant shifts in capacity. Several key genes are absent from glycolysis (Embden-Meyerhof-Parnas; EMP), although both strains have retained key genes in the alternative Entner-Doudoroff (ED) pathway, allowing for catabolism of glucose to glyceraldehyde-3-phosphate via this alternate mechanism (Fig. 3). Distinct from the capabilities of 868, strain 880 possesses a complete standard ED pathway (Fig. 3). The TCA cycle has lost approximately half of the genes in both strains, resulting in an inability to convert succinyl-CoA into succinate or any of the downstream metabolites (Additional file 2: Fig. S2). The fate of succinyl-CoA is likely tied to lysine biosynthesis via the diaminopimelate (DAP) pathway and/or glycine-mediated heme biosynthesis via synthesis of 5-aminolevulinic acid (ALA) from glycine and succinyl-CoA catalyzed by aminolevulinic acid synthase (ALAS) [22]. Additionally, both strains uniquely possess several genes for thiamine synthesis (Fig. 2). Whole genome alignment produced 12 locally collinear blocks (LCBs; Fig. 1) revealing several shifts in orientation and location within the genome of each strain. There were 1598 shared genes between the strains.

### Horizontal gene transfer and functional profiles of gut associates

Widespread horizontal gene transfer has had a profound effect on the genomes of both AAB strains. We employed several precautions to ensure sterility during culturing (see Methods). As an extra step, we also aligned all Illumina reads from isolate sequencing to the closed genomes. We detected highly even coverage of Illumina reads across the genome, as expected for isolate bacterial genomes (Additional file 3: Figure S3 and Additional file 4: Figure S4), as compared to mixed communities or contaminated cultures which would yield variable coverage of genes from contaminant organisms.

**Fig. 4:**
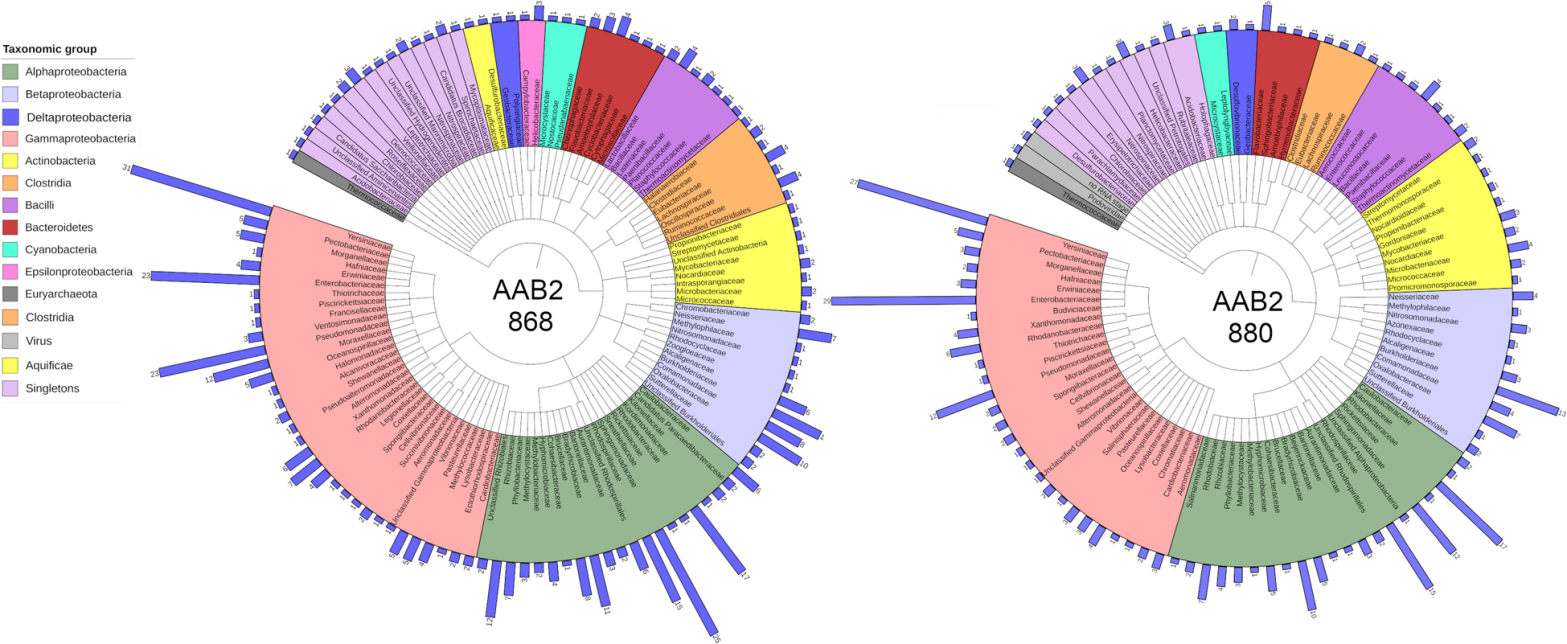
Genomic content of isolates by phylogenetic distribution of genes. Families are colored by the taxonomic group listed. Blue bars indicate the number of genes acquired from the Family listed; numbers at the end of each bar indicate the count acquired from each group

**Fig. 5:**
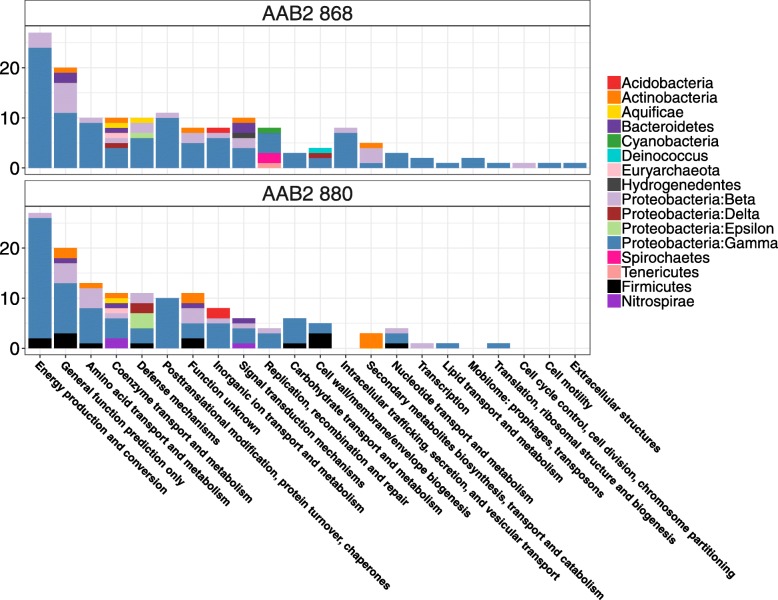
Horizontally transferred genes by phylogenetic origin and functional classification. Genes acquired via HGT are enumerated according to associated COG and colored by taxonomic classification

The total count of horizontally transferred genes within the genomes of strains 868 and 880 was 342 and 276, which amounted to 16.2 and 14.73%, respectively, and spanned several phyla (Table 1, Fig. 4). Because we employed a criterion more conservative than the default for identifying horizontally transferred genes, our analyses are likely biased toward genes from recent transfer events. Genes acquired less recently and/or those with reduced selective pressure were likely to be missed by our threshold. The functional profile of horizontally transferred genes in 868 and 880 was diverse and spanned several COG categories and multiple phyla (Fig. 5). The counts of horizontally transferred genes across COG categories ranged from one to ~ 30. The COG functional category with the greatest extent of HGT was “Energy production and conversion” and included acquisition of all 14 subunits of NADH dehydrogenase from Gammaproteobacteria (potentially from the Enterobacteraceae) origin (Fig. 5, Additional file 6: Figure S6). Gammproteobacteria was the taxonomic class with the greatest contribution of horizontally transferred genes, and the Rhizobiales were the family with greatest contribution. Across both strains, “Coenzyme transport and metabolism” was the COG category with the greatest diversity of phyla. Comparative Pfam profiles (Additional file 7: Figure S7) of various AAB taxa suggested that both strains of AAB2 showed significant (*P* < 0.0001) enrichment of Sel1 domains (Additional file 10: Table S1). Strains 868 and 880 also showed significant (*P* < 0.0001) enrichment of LysE type translocators implicated in the extracellular export of L-lysine [23]. Both of these PFam domains are almost entirely absent in other AAB taxa, suggesting that their enrichment is unique to isolates 868 and 880. Conversely, both isolates also displayed a reduction in phage integrase family proteins, a family that cleaves DNA and aids in recombination [24] (Additional file 7: Figure S7).

### Phylogenomic inference

**Fig. 6:**
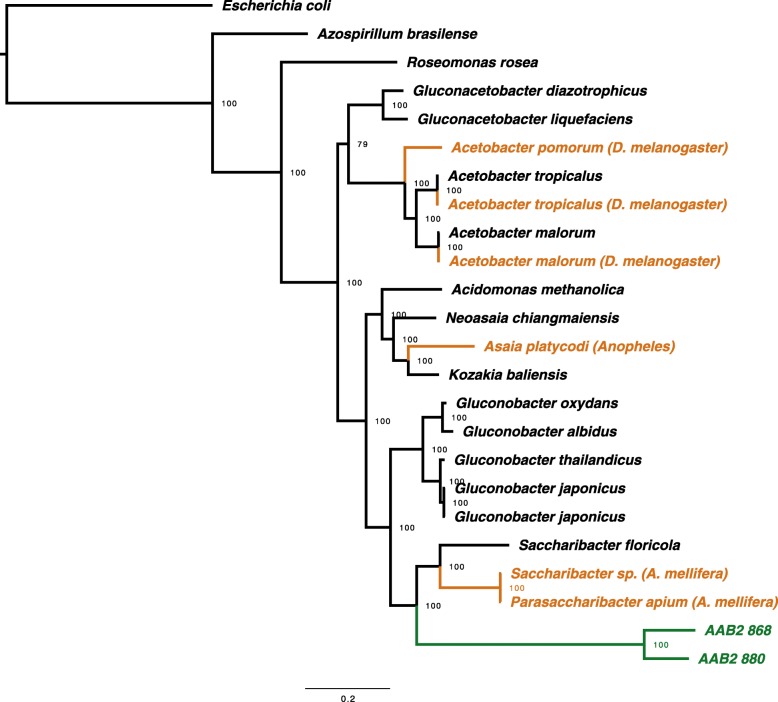
Phylogenetic analysis of AAB from diverse sources. Bayesian tree showing the phylogenetic positions of both strains described here relative to other members of the Acetobacteraceae. Evolutionary relationships were reconstructed using amino acid sequences from ten single-copy orthologs. Clade credibility values were generated from 1,000,000 MCMC generations. Ant associates fully sequenced in this study are colored green, other insect associated bacterial isolates are colored orange, and environmental isolates are colored black. Hosts are indicated in parentheses, where applicable (D. melanogaster: *Drosophila melanogaster;* A. mellifera: *Apis mellifera*). Bar, 0.2 expected changes per site

Phylogenomic inference revealed deep divergence of both AAB strains (Fig. 6). In line with previous analyses of AAB1 and AAB2 lineages based on 16S rRNA [12], our estimations placed both AAB2 strains in a deeply divergent, monophyletic clade with perfect bootstrap support, suggesting genus-level divergence or greater. Distinct from previously published 16S rDNA based trees [12], which are likely to underestimate genomic diversity, Bayesian inference from amino acid sequence data strongly suggested that AAB2 and its relatives in bees are sister to *Gluconobacter* species.

## Discussion

From large-scale genomic erosion [25] to host specialization [7, 10], symbiotic associations have profound effects on the evolution of bacterial associates. Our results shed light on processes shaping genomes of endogenous bacterial associates of camponotine ants. Compared to free living AAB, AAB2 strains 868 and 880 possess genomes reduced in both size and biosynthetic capacity. Genome reduction in bacterial associates of insects is well documented and most evident in ancient symbioses with intracellular mutualists [26]. Recently, several studies have demonstrated that to a smaller extent, extracellular gut associates may undergo similar processes, resulting in large scale gene loss and alterations in metabolic capacity [2, 7, 25, 27, 28]. Our findings of mean reductions in genome size by ~ 1.2 MB and GC content by ~ 15% (compared to free living relatives) are consistent with changes seen in other extracellular gut associates [2, 7, 25] with long coevolutionary histories [29].

Horizontal gene transfer, a hallmark of the gut as an ecological niche [30], has had a profound impact on the content and functional potential of both AAB2 strains. Approximately 15% (Table 1) of the genome of each AAB strain was acquired via horizontal transfer from closely related taxa, as well as across phyla and kingdoms (Fig. 4). Because we used a more conservative criterion for calling horizontally transferred genes than generally used, our selection favors genes acquired via more recent transfer. COG functional profiles of horizontally transferred genes (Fig. 5) suggest that acquisition was non-uniform across functional categories, with genes associated with energy production and conversion having the highest incidence of acquisition, a finding recently reported for HGT between bacteria and archaea in anaerobic or high temperature environments [31]. This may be due to a cascade effect in which shifts in metabolism or substrate availability may require an expanded energy investment into salvage pathways or metabolite conversion. In such cases, an increased repertoire of energy production and conversion pathways would lead to increased fitness of symbionts with such production plasticity. In both AAB2 strains, the remarkable acquisition of the complete NADH-quinone oxidoreductase complex (Additional file 6: Figure S6) would increase the capacity for H+ export to create a proton gradient necessary for ATP synthesis via oxidative phosphorylation (OP).

As a whole, central metabolism and OP represent highly mosaic processes in both strains. Loss of individual enzymes from metabolic cycles has been documented in certain gut associates [7, 25, 29], with mutualists of social bees utilizing an alternate enzyme during the TCA cycle to catalyze succinyl-CoA - > succinate conversion [32]. However, our results suggest that several key metabolic pathways suffer losses of up to 50% of key enzymes. Both AAB2 isolates have lost the initial half of the genes required for the standard EMP pathway of glycolysis (Fig. 3), as well as the latter half of the TCA cycle, breaking its cyclical structure (Additional file 2: Fig. S2). The decrease in fitness associated with the loss of these genes is likely partially offset via the retention of select genes (though not all) in the ED and OPP pathways, resulting in an atypically mosaic approach to the metabolism of glucose to glyceraldehyde-3-phosphate (G3P; Fig. 3). Specifically, both strains lack genes encoding the first five reactions of the EMP pathway, whereby glucose is converted to G3P. However, after the phosphorylation of glucose by glucokinase (Glk), glucose-6P (G6P) appears to be converted to gluconate-6P by G6P dehydrogenase (Zwf) and 6-phosphogluconolactonase (Pgl), both canonical enzymes in the OPP. Interestingly, a key downstream enzyme in the OPP, ribulose-phosphate 3-epimerase (Rpe), which converts ribulose-5P to xylulose-5P, is missing, leaving both strains without the ability to generate G3P solely by the OPP. This suggests that phosphogluconate dehydratase (Edd) and 2-Keto-3-deoxy-6-phosphogluconate aldolase (Eda), canonical enzymes in the ED pathway, represent the most straightforward glycolytic route to G3P. G3P is the first metabolite able to be processed by the truncated EMP glycolytic cycle retained in 868 and 880, thereby completing glucose metabolism through subsequent conversion to pyruvate.

Although genomic erosion has reduced, but not eliminated glycolytic routes in both strains, the cyclical nature of the TCA cycle seems to have been completely broken (Additional file 2: Figure S2). All enzymes needed to catalyze reactions after the production of succinyl-CoA have been lost. This is noteworthy, as the next reaction in the cycle is the conversion of succinyl-CoA to succinate, which generates one molecule of ATP or GTP. While empirical evidence for the fate of succinyl-CoA is needed, our results suggest two possible endpoints: as a carbon donor in glycine-mediated heme biosynthesis, or as a component in N-succinyl-L-2-amino-6-oxopimelate synthesis during the DAP pathway of L-lysine biosynthesis. Broadly, heme biosynthesis can be broken down into three key parts [33]: (i) precursor formation (5-aminolevulinic acid; ALA), (ii) generation of the cyclic tetrapyrrole, uroporphyrinogen III, and (iii) conversion into heme. Both AAB2 strains retain the gene for 5-aminolevulinic acid synthase (ALAS), which produces ALA from a condensation reaction between glycine and succinyl-CoA, and both are glycine prototrophs [22, 33]. The genomes of both strains possess all of the enzymes required for step 2, synthesis of uroporphyrinogen III, but neither possess the typical analog for protoporphyrinogen oxidase (protox) (Additional file 8: Figure S8). Conversion of protoporphyrinogen IX to protoporphyrin IX by protox is the penultimate step in bacterial heme synthesis from ALA and is encoded by the hemG gene in bacteria [33, 34]. However, Kato et al. [35] identified a novel protox enzyme, hemJ, which has an analog in the genomes of both stains, completing the pathway for heme synthesis from ALA and glycine [34] (Additional file 8: Figure S8). Additionally, succinyl-CoA is a key component of L-lysine biosynthesis via the DAP pathway [36] by 2,3,4,5-tetrahydropyridine-2, 6-dicarboxylate N-succinyltransferase, which is present in both strains. This option fits well with the observed conservation of L-lysine biosynthesis pathways and the high abundance of L-lysine transporters. Therefore, we hypothesize that heme and lysine biosynthesis ameliorate accumulation of succinyl-CoA from the broken TCA cycle.

Across both strains, several pathways for the biosynthesis of amino acids and precursor molecules have undergone substantial changes (Fig. 2). Neither AAB strain is predicted to be able to synthesize chorismate, the precursor molecule to aromatic amino acid synthesis, or any of the aromatic amino acids. Compared to other acetic acid bacteria, AAB2 strains have lost genes in biosynthetic or conversion pathways of histidine, leucine, methionine, and arginine, although both have acquired genes for the synthesis or conversion of cysteine and serine. Additionally, both AAB2 isolates have acquired genes in pathways for thiamine and derivative synthesis and regeneration, molybdenum cofactor synthesis, and CoA biosynthesis.

Furthermore, both display significant enrichment of LysE type transporters (*P* < 0.0001) and Sel1-like repeat (SLR; *P* < 0.0001) proteins compared to other non ant associated Acetobacteraceae (Additional file 7: Figure S7; Additional file 10: Table S1). LysE type translocators are membrane spanning L-lysine and L-arginine exporters that have been empirically shown to excrete both amino acids into extracellular space in *C. glutamicum* [37]. In addition to enrichment of LysE type transporters (Additional file 7: Figure S7), both AAB2 strains have retained several pathways associated with lysine synthesis and conversion. The acquisition and maintenance of multiple lysine exporters is suggestive of a physiological role in the relationship between AAB2 isolates and *C. chromaiodes:* supplementation of L-lysine. The diet of camponotine ants is variable, including plant detritus and the carcasses of other insects. Due to this diversity, it is difficult to speculate how patterns of nutrient availability may shape ecological networks within the gut tract. However, previous work has demonstrated host nutritional upgrading by the camponotine gut endosymbiont, *Blochmannia* [38], suggesting that nutritional contributions from bacterial metabolism may help stabilize nutrient availability in the gut. The genome of the ancient *Camponotus* endosymbiont, *Blochmannia,* [39, 40] has been shown to retain biosynthetic pathways for amino acids utilized by the host [41]. In gut associated bacteria, where reductive evolution acts rapidly to deplete functionality of nonessential genes [25, 29], enrichment and maintenance of loci is strongly suggestive of functionality. Notably, both isolates have retained biosynthetic capacities for L-lysine via several pathways (Fig. 2).

SLR proteins possess diverse roles in cellular processes including serving as adaptor proteins in supermolecular complexes. They have also been implicated in the cellular stress response and in mediating interactions between host and bacterial cells [42]. In *Helicobacter pylori,* enrichment and positive selection of SLR proteins has been implicated in coevolution with innate immune enzymes [43] and proposed to facilitate the maintenance of chronic infection [44]. Enrichment of SLR proteins across the genomes of both isolates (Additional file 7: Figure S7) may be symptomatic of a long-term relationship with *Camponotus*.

Aside from genomic plasticity due to gene gain and loss, persistent association with a host also directs selective pressures across the genome. We performed phylogenetic analyses of each isolate to estimate divergence from other host associated and free living bacteria (Fig. 6). In accord with phylogenetic reconstructions based on 16S rRNA [12], both strains of AAB2 formed a deeply divergent, monophyletic clade that was sister to gut associated *Saccharibacter* in *A. mellifera*. Additionally, our results robustly placed the AAB2 and *Saccharibacter* taxa sister to *Gluconobacter* species with perfect clade credibility from posterior probabilities.

Plasmids, which often encode locally adaptive traits, were detected in strain 868 but not 880, though it is unclear if this was due to biological or technical reasons. We did not use plasmid-specific kits for gDNA isolation, which may have biased our results. Furthermore, short DNA fragments (< 10 kb) are typically discarded during library preparation for PacBio sequencing and this may bias against shorter plasmid sequences. It is also possible that isolate culturing may have eliminated or reduced plasmid copy numbers, as evident by the low copy plasmid (via PacBio coverage) detected in strain 868 (though Illumina sequence alignment suggested comparable levels to chromosomal DNA; Additional file 3: Figure S3, Additional file 4: Figure S4 and Additional file 5: Figure S5). The single plasmid detected in strain 868 encoded 32 predicted genes. Other than recombinase and mobilization proteins, most genes were predicted to produce hypothetical proteins or those with general function prediction only. However, we did detect two genes predicted to encode Type VI valine-glycine repeat proteins (VgrG). VgrG proteins have recently been shown to elicit the secretion and antibacterial activity of type VI DNase toxins [45], potentially to modulate interbacterial competition. We also detected a type II toxin-antitoxin system, which may facilitate maintenance of the plasmid in strain specific populations [46]. It appears likely that the strain 868 plasmid confers a fitness advantage by reducing competition with other gut bacteria.

We attempted to culture all lineages within Acetobacteraceae that have been previously associated with *Camponotus* hosts [12]. However, despite detecting members of the AAB1 lineage in multiple host colonies, we were unable to culture them under the conditions that were conducive to both AAB2 strains. Whether our inability to culture AAB1 was due to stricter or altered metabolic requirements remains unknown, but previous analyses of the 16S structure suggest greater destabilization and, potentially, longer host association and/or greater host dependence [12].

Collectively, our results delineate ecological and evolutionary factors associated with bacterial adaptation to the gut environment. While hallmarks such as genomic erosion and loss of metabolic potential are well documented in reliable gut associates, our data are the first to delineate both genomic erosion and extensive, genome-wide horizontal gene acquisition, resulting in gut-specific strains with mosaic genomes (Figs. 3 and 4). Elevated levels of horizontal transfer of genomic content have been previously reported in the gut environment [30], and our data illustrate patterns of extensive transfer and acquisition of genetic material between closely related taxa and across phyla. Comprising approximately 15% of protein coding sequences in each AAB strain (Table 1), horizontally transferred genes have drastically increased the metabolic and biosynthetic potential of each isolate, potentially offsetting the patterns of reductive evolution germane to gut adaptation. Due to these two processes, genomes of gut associated AAB have rapidly diverged from closely related environmental isolates. Together, our results illustrate processes facilitating colonization of and adaptation to the gut environment by bacterial associates.

## Methods

### Ant collection and sample preparation

Colonies of *Camponotus chromaiodes* were collected from Duke Forest, Durham, NC and Durant Nature Preserve, Raleigh NC during October and November 2016. *C.chromaiodes* was chosen due to its high abundance in North Carolina and results of previous work suggesting potential host adaptation [12]. Based on the prevalence of AAB1 and AAB2 among the worker caste [12], only minor and major workers were collected from each colony. All specimens were collected alive and transported to Duke University immediately. Prior to downstream processing, specimens were anesthetized by placing in 15 mL test tubes and immersing in ice.

Prior to dissection, all anesthetized ants were surface sterilized. Samples were sterilized by immersion in 100% ethanol, followed by a 60 s soak in a 5% bleach solution, and finally rinsed in sterile water. Upon surface sterilization, the entire alimentary tract (fore-, mid-, and hindgut) was dissected out under a microscope. Dissected tissue samples were pooled in sterile PBS and held on ice until homogenization. Pooled tissue samples were lightly homogenized with a plastic pestle and handheld homogenizer.

### Bacterial isolate culturing

Prior to inoculation, all culture plates and broths were allowed to de-gas for 24 h at room temperature in an anaerobic chamber with an environment consisting of 5% H, 5% CO_2_, 90% N. All bacterial cultures were sterilely plated, passaged, and/or inoculated under these conditions, then placed in a GasPak EZ (BD) anaerobic container and transferred to an incubator held at 29’C under the same environment. Homogenized gut tracts suspended in sterile PBS were directly plated onto MRS agar (Hardy Diagnostics) for the growth and selection of acetic acid bacteria and left to incubate for 2–5 days, or until colonies appeared. MRS agar was chosen because it has been successfully used to culture Acetobacteraceae from insect guts [47] and yielded positive growth in our experiments. Prior to isolation, bacterial colonies were screened with strain-specific primers as described below. Colonies with positive amplification from strain-specific primers were isolated on replica plates and streaked to isolate populations arising from a single cell. Colonies arising from streaked individual cells were transferred to sterile liquid MRS broth and incubated at 29’C under the same environment for 2–5 days. Glycerol stocks of all cultures were diluted to 25% glycerol and stored at − 80’C.

### PCR screens, gDNA extraction, and Sanger sequencing

Prior to isolate plating and growth in liquid culture, bacterial colonies grown from tissue homogenate were screened using primers specific to 16S rRNA of *Camponotus* AAB2, as described previously [12]. Colony PCR was performed using the Phusion High-Fidelity Master Mix with HF Buffer (NEB). PCR programs were held at 98 °C for 30 s to denature the DNA, followed by 30 cycles of amplification proceeding at 98 °C for 10s, 61 °C for 30s, and 72 °C for 60s, a final extension phase was held at 72 °C for 5 min, then cooled at 4 °C. Colonies with positive amplification were isolated on replica plates and streaked for isolation. Pure liquid cultures were centrifuged at 5000×g for 10 min and gDNA was extracted from cell pellets using the Qiagen (Hilden, Germany) DNEasy kit. gDNA was extracted from tissue samples using the same kit. We used the standard protocol for animal tissue for dissected gut tracts and the recommended gram-negative pretreatment for cell cultures of acetic acid bacteria. To check that our liquid cultures remained free of contamination during culturing, we performed Sanger sequencing with universal 16S primers on gDNA extracted from cell pellets. PCR for Sanger sequencing was performed using the Phusion High-Fidelity Master Mix with HF Buffer (NEB). PCR programs were held at 98 °C for 30 s to denature the DNA, followed by 30 cycles of amplification proceeding at 98 °C for 10s, 55 °C for 30s, and 72 °C for 60s, a final extension phase was held at 72 °C for 5 min, then cooled at 4 °C. Universal primers 16S 9F (5′ GAGTTTGATCCTGGCTCA ′3) and 16S 1507R (5’TACCTTGTTACGACTTCACCCCAG ‘3) were used to identify any contaminating 16S sequences with BigDye chemistry. All products were sequenced on an ABI 3730xl (Life Technologies, Carslbad, CA, USA) at Duke University.

### Bacterial isolate full genome sequencing, assembly, and analysis

Approximately 15μg of gDNA from each sample was used for full genome sequencing. Large insert library preparation and sequencing was performed by the Genomic and Computational Biology Core Facility at Duke University. Bacterial isolate genomes were sequenced on a PacBio RSII (Pacific Biosciences), utilizing one SMRT cell per isolate. De novo genome assembly was performed using the HGAP assembler [48] and resulted in fully closed genomes for both strains. Genomic summary statistics are listed in Table 1. Isolate genomes were also separately sequenced on an Illumina HiSeq 2500 platform. Illumina library prep and 150PE sequencing was performed by the Genomic and Computational Biology Core Facility at Duke University. Illumina reads were not used in assembly, but were aligned to the closed genome and plasmid sequences generated on the PacBio RSII to examine genomic coverage. Reads were aligned using Bowtie2 [49] and positional mapping information was extracted using SAMtools [50].

Closed bacterial genomes were uploaded to the Joint Genome Institute (JGI) Integrated Microbial Genomes and Microbiomes (IMG) analysis server and database. Gene prediction and annotation were performed using the JGI Microbial Genome Annotation Pipeline [51]. Identification of tRNA and rRNA genes and CRISPR elements was performed using the IMG Expert Review system Standard Operating Procedure [52]. KEGG Orthology terms and Pathways, COG function, Pfam assignment, and IMG Network Reconstruction were performed using IMG’s automated toolkit [52]. Whole genome alignment and visualization was performed with the Mauve aligner [53] on nucleotide sequence data, using the progressiveMauve algorithm within Mauve [53]. All statistical analyses were performed in R. Gene set statistical analysis was performed using Fisher’s Exact test. The vegan, ape, and ggplot2 packages were used for data manipulation and visualization [54–56].

#### Pseudogene analysis

Pseudogenes were detected using LAST [57] within the DFAST framework [58]. Briefly, CDS sequences and flanking regions were locally re-aligned to the subject sequence using BLAST. Sequences with stop codons, frameshifts, or potential alternate amino acids (eg. selenocysteine, pyrrolysine) in the flanking regions were identified as possible pseudogenes.

### Determination of horizontally transferred genes

We used the standard protocol of the IMG comparative analysis system to detect horizontally transferred genes [52], although we applied a stricter criterion for horizontal transfer than the default pipeline. Briefly, protein coding gene sequences were identified using the Prodigal v2.50 ab initio gene prediction program [59]. Translated nucleotide sequences were queried against the NCBI Protein Reference Sequence Database using default parameters and the best BLAST hit and associated taxonomy were extracted. We manually removed hits with e-values below 1e^− 5^. COG and KOG assignment was performed using RPS-BLAST against the NCBI Conserved Domain Database [60], using a BLAST cutoff of 1e^− 5^ and requiring that the alignment length spanned 70% of the reference sequence length.

### Phylogenomic inference

#### Selection of taxa

In total, 24 taxa were selected for Bayesian phylogenetic inference, 22 of which were members of the Acetobacteraceae. Taxa were chosen on the basis of maximizing the breadth of genomic diversity within the Acetobacteraceae while weighting closely related taxa more heavily. Where possible, we chose isolates associated with insect guts as well as environmental isolates of the same species. Included genomes were restricted to those that were marked as high-quality drafts or finished assemblies.

Homologs shared among all taxa used in phylogenetic inference were determined using IMG’s analysis pipeline. Single copy orthologs were identified using OrthoMCL [61], and amino acid sequence alignment was performed using Clustal Omega [62]. Ambiguously aligned regions were removed using Gblocks [63] though constraints were slightly relaxed to allow for smaller final blocks, and to allow less stringent flanking positions. These criteria were used to maximize the length of the multiple sequence alignment and were manually inspected to be of high alignment quality. The final alignment spanned 3314 unambiguously aligned amino acid positions over 10 single-copy genes (Additional file 9: File S1). We used MrBayes v3.2.1 [64] for Bayesian phylogenetic inference of the aligned amino acid sequences, employing a General Time Reversible (GTR) model with a Gamma (Γ) distribution of rate heterogeneity [65] and proportion of invariant site estimation. Clade credibility was derived from posterior probability values generated from 1,000,000 MCMC generations with a relative burnin percentage of 25%. The average deviation of split frequencies was low at the end of the run (< 0.0001), suggesting that the two runs had converged onto a stationary distribution.

## Additional files

Additional file 1: **Figure S1.** Functional Profiles by COG category of both AAB2 strains sequenced in this study and other host associated and free living isolates. Hosts are indicated in parentheses, where applicable (Dm: *Drosophila melanogaster; Asp: Anopheles* species; Am: *Apis mellifera*). (PDF 94 kb)

Additional file 2: **Figure S2.** The TCA cycle in AAB2 strains 868 and 880. Genes present in both strains are colored purple. (TIFF 93 kb)

Additional file 3: **Figure S3.** Illumina read coverage across the genome of isolate AAB2 868. (TIFF 2152 kb)

Additional file 4: **Figure S4.** Illumina read coverage across the genome of isolate AAB2 880. (TIFF 2152 kb)

Additional file 5: **Figure S5.** Illumina read coverage across the plasmid of isolate AAB2 868. (PDF 1250 kb)

Additional file 6: **Figure S6.** Oxidative Phosphorylation pathways in both AAB2 strains sequenced in this study. Genes present in both strains are colored purple and those present in one strain are colored orange. The complete NADH-quinone oxidoreductase complex (*nuo*) was acquired via HGT and is outlined in red. (PNG 160 kb)

Additional file 7: **Figure S7.** Abundance of PFam annotations across the genomes of several free living and host associated AAB. The ten most abundant function categories within the genomes of AAB isolates as annotated by PFam category. Labels of ant associates fully sequenced in this study are colored green, other host associated bacterial isolates are colored orange, and environmental isolates are colored black. Hosts are indicated in parentheses, where applicable (Dm: *Drosophila melanogaster; Asp: Anopheles* species; Am: *Apis mellifera*). (PDF 6 kb)

Additional file 8: **Figure S8.** Heme biosynthesis in AAB2 strains 868 and 880. Genes present in both strains are colored purple. (PNG 168 kb)

Additional file 9: **File S1.** Amino acid alignment used for Bayesian phylogenomic analysis. (FASTA 86 kb)

Additional file 10: **Table S1.** Significantly differentially abundant Pfam categories from gene set enrichment analysis of AAB2 strains against other Acetobacteraceae (XLS 32 kb)

## Data Availability

The complete genome data sets supporting the results of this article are available in the JGI GOLD database under analysis project IDs Ga0173700 and Ga0174704, in IMG/MER under submission IDs 115725 and 115788, and in GenBank under BioProject PRJNA532976. For all colonies, ant vouchers (in the form of whole samples or dissected remains) are stored at Duke University and are available to view upon request.

## Abbreviations

AAB: Acetobacteraceae
ALA: 5-aminolevulinic acid
ALAS: Aminolevulinic acid synthase
COG: Cluster of Orthologous Groups
ED: Entner-Doudoroff
Eda: KDPG aldolase
Edd: Phosphogluconate dehydratase
EMP: Embden-Meyerhof-Parnas
Fba: Fructose bisphosphate aldolase
Gdh: Glucose dehydrogenase
GDL: Glucono delta-lactone
Glk: Glucokinase
Gnd: 6-phosphogluconate dehydrogenase
Gnl: Gluconolactonase
GntK: Gluconokinase
GTR: General Time Reversible
IPWAY: IMG Pathway
LAB: Lactobacillaceae
MCMC: Markov chain Monte Carlo
OP: Oxidative Phosphorylation
OPP: Oxidative Pentose Phosphate Pathway
Pfam: Protein family
Pfk: 6-phosphofructokinase
Pgi: phosphoglucose isomerase
Pgl: 6-phosphogluconolactonase
Rpe: Ribulose-5-phosphate
RpiA: Ribose-5-phosphate isomerase A
SLR: Sel-1 Like Repeat
TCA: Tricarboxylic Acid cycle
TktA: Transketolase 1
VgrG: Type VI valine-glycine repeat proteins
Zwf: Glucose-6-phosphate dehydrogenase
