# Supplementary figures and images for: Genomic erosion and extensive horizontal gene transfer in gut-associated Acetobacteraceae
Source: BMC Genomics. 2019 Jun 10;20:472. doi: 10.1186/s12864-019-5844-5 (PMC6558740; doi:10.1186/s12864-019-5844-5)

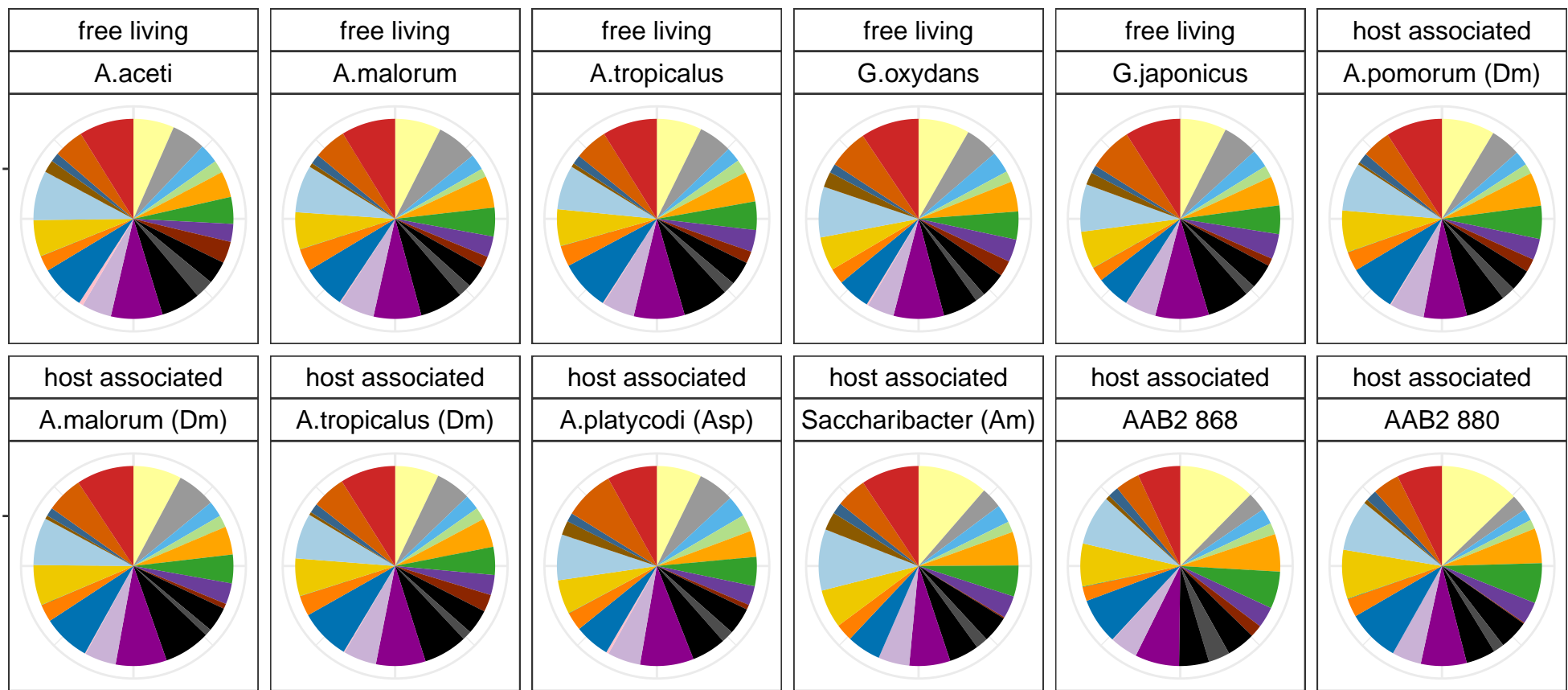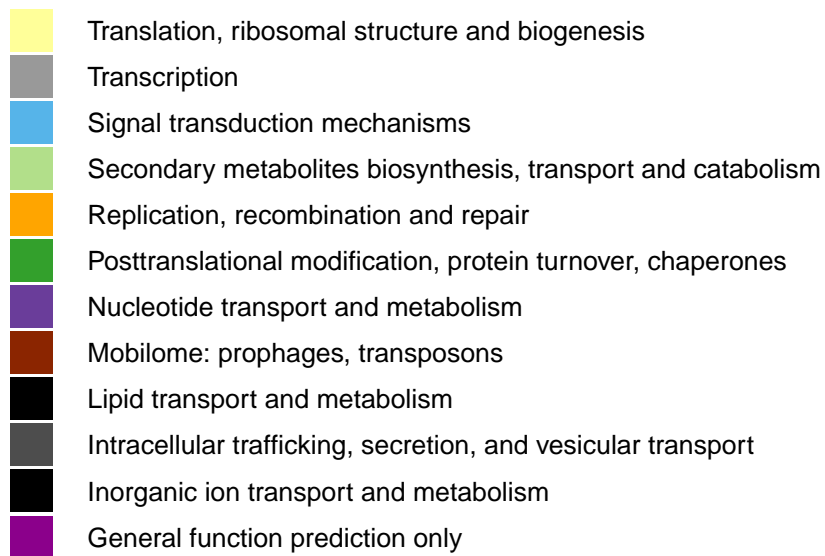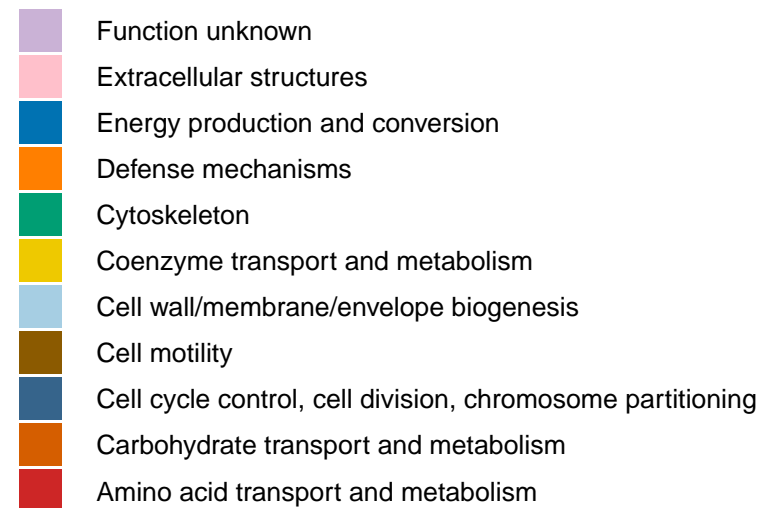

COG

Supplement: Supplementary file 1 — Figure S1. Functional Profiles by COG category of both AAB2 strains sequenced in this study and other host associated and free living isolates. Hosts are indicated in parentheses, where applicable (Dm: Drosophila melanogaster; Asp: Anopheles species; Am: Apis mellifera). (PDF 94 kb) [file 12864_2019_5844_MOESM1_ESM.pdf]

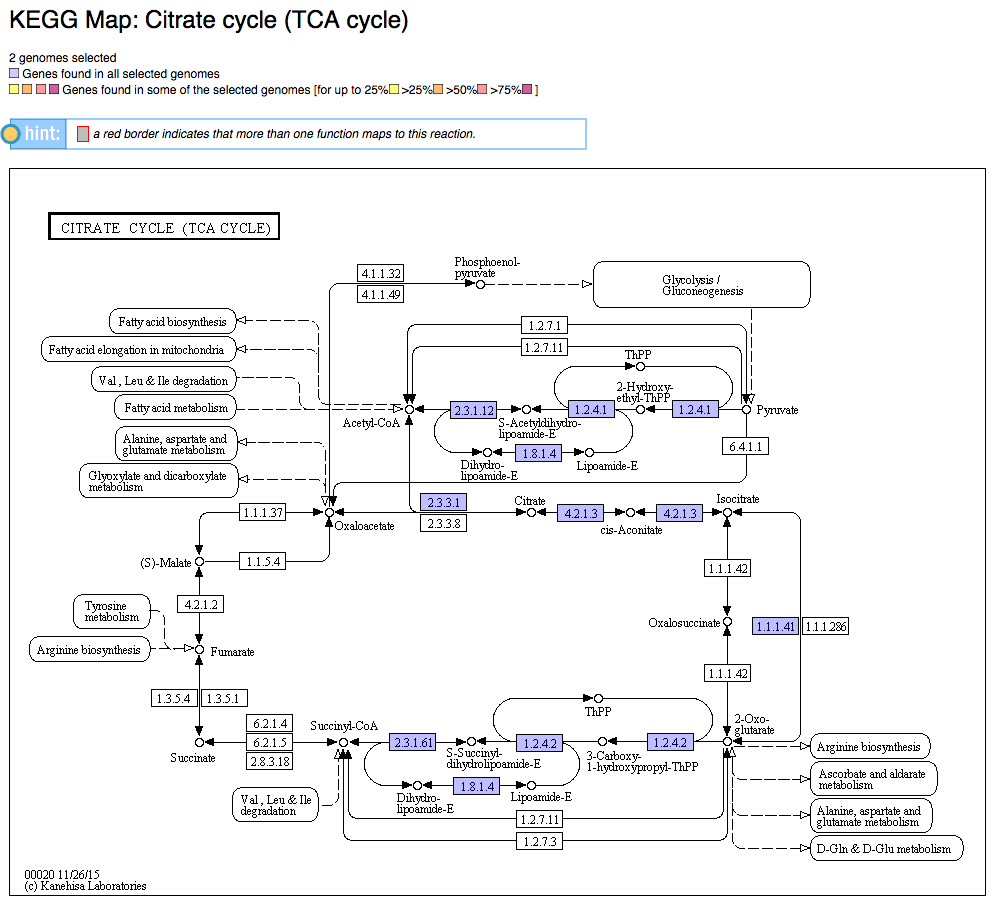

Supplement: Supplementary file 2 — Figure S2. The TCA cycle in AAB2 strains 868 and 880. Genes present in both strains are colored purple. (TIFF 93 kb) [file 12864_2019_5844_MOESM2_ESM.tiff]

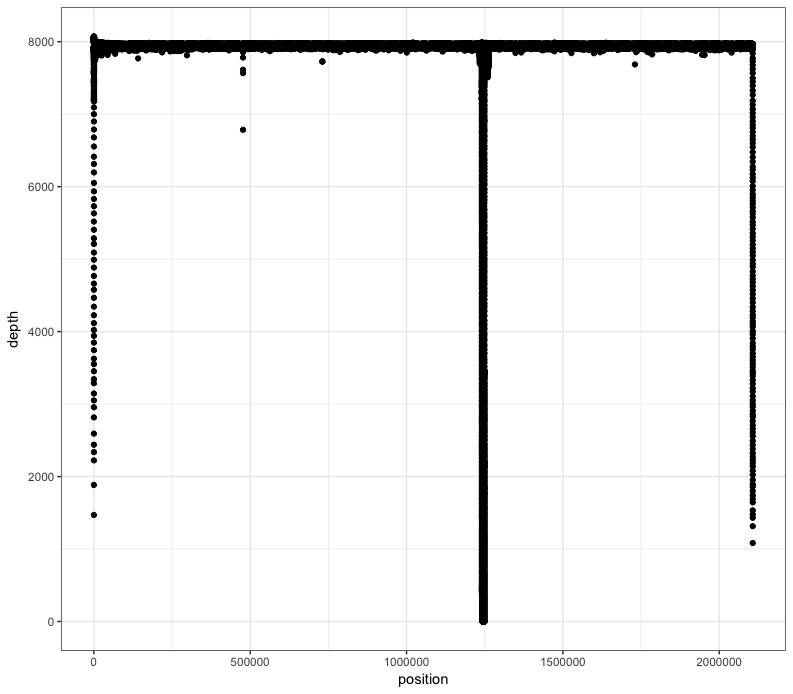

Supplement: Supplementary file 3 — Figure S3. Illumina read coverage across the genome of isolate AAB2 868. (TIFF 2152 kb) [file 12864_2019_5844_MOESM3_ESM.tiff]

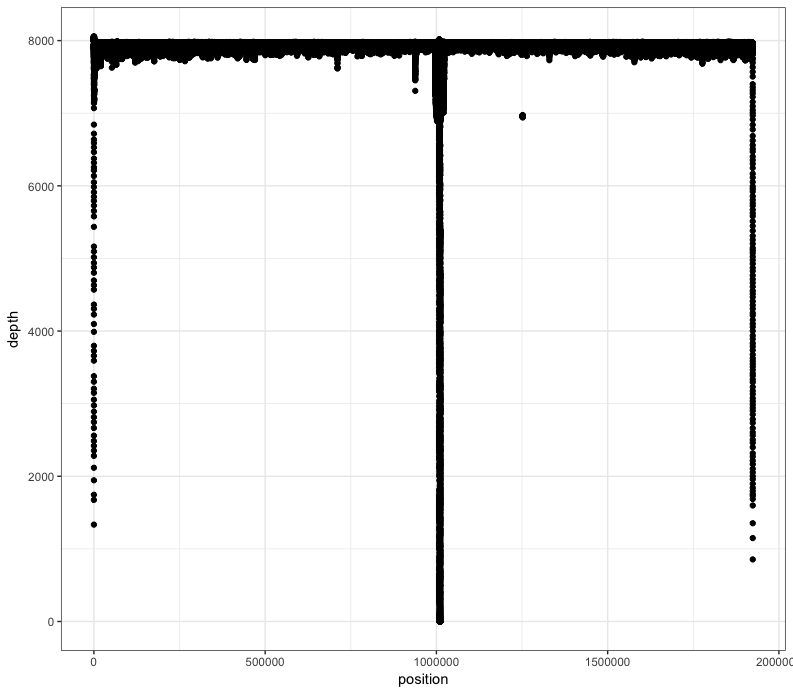

Supplement: Supplementary file 4 — Figure S4. Illumina read coverage across the genome of isolate AAB2 880. (TIFF 2152 kb) [file 12864_2019_5844_MOESM4_ESM.tiff]

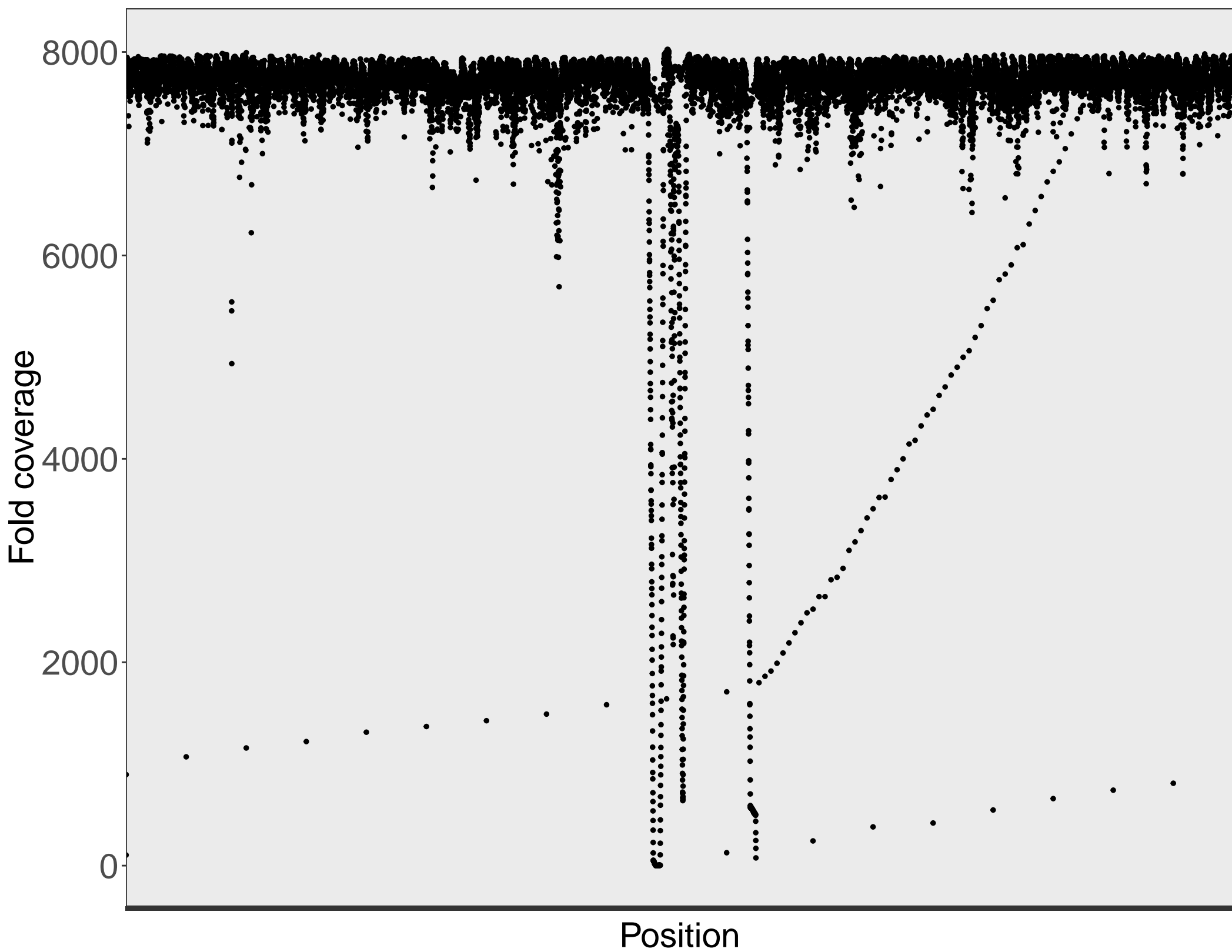

Supplement: Supplementary file 5 — Figure S5. Illumina read coverage across the plasmid of isolate AAB2 868. (PDF 1250 kb) [file 12864_2019_5844_MOESM5_ESM.pdf]

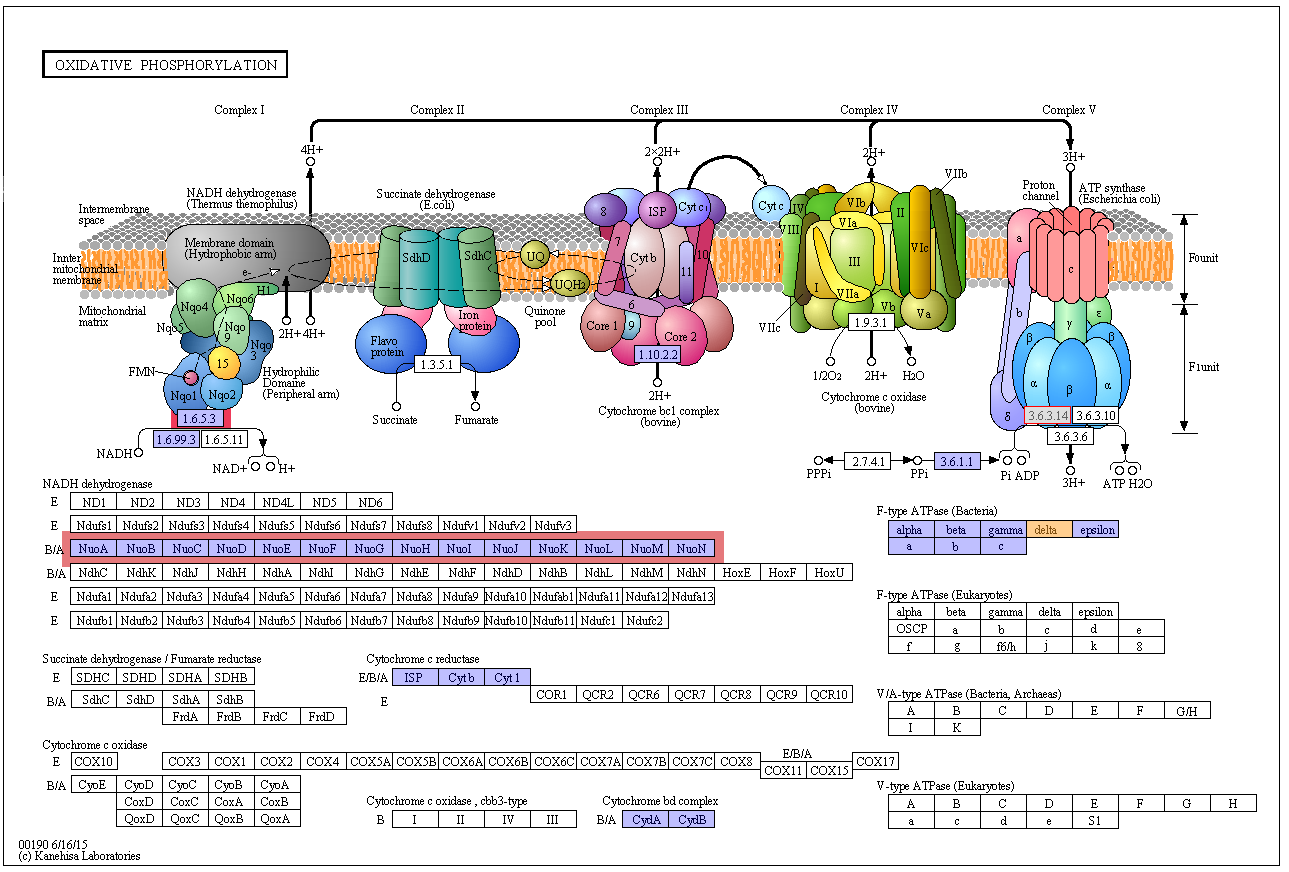

Supplement: Supplementary file 6 — Figure S6. Oxidative Phosphorylation pathways in both AAB2 strains sequenced in this study. Genes present in both strains are colored purple and those present in one strain are colored orange. The complete NADH-quinone oxidoreductase complex (nuo) was acquired via HGT and is outlined in red. (PNG 160 kb) [file 12864_2019_5844_MOESM6_ESM.png]

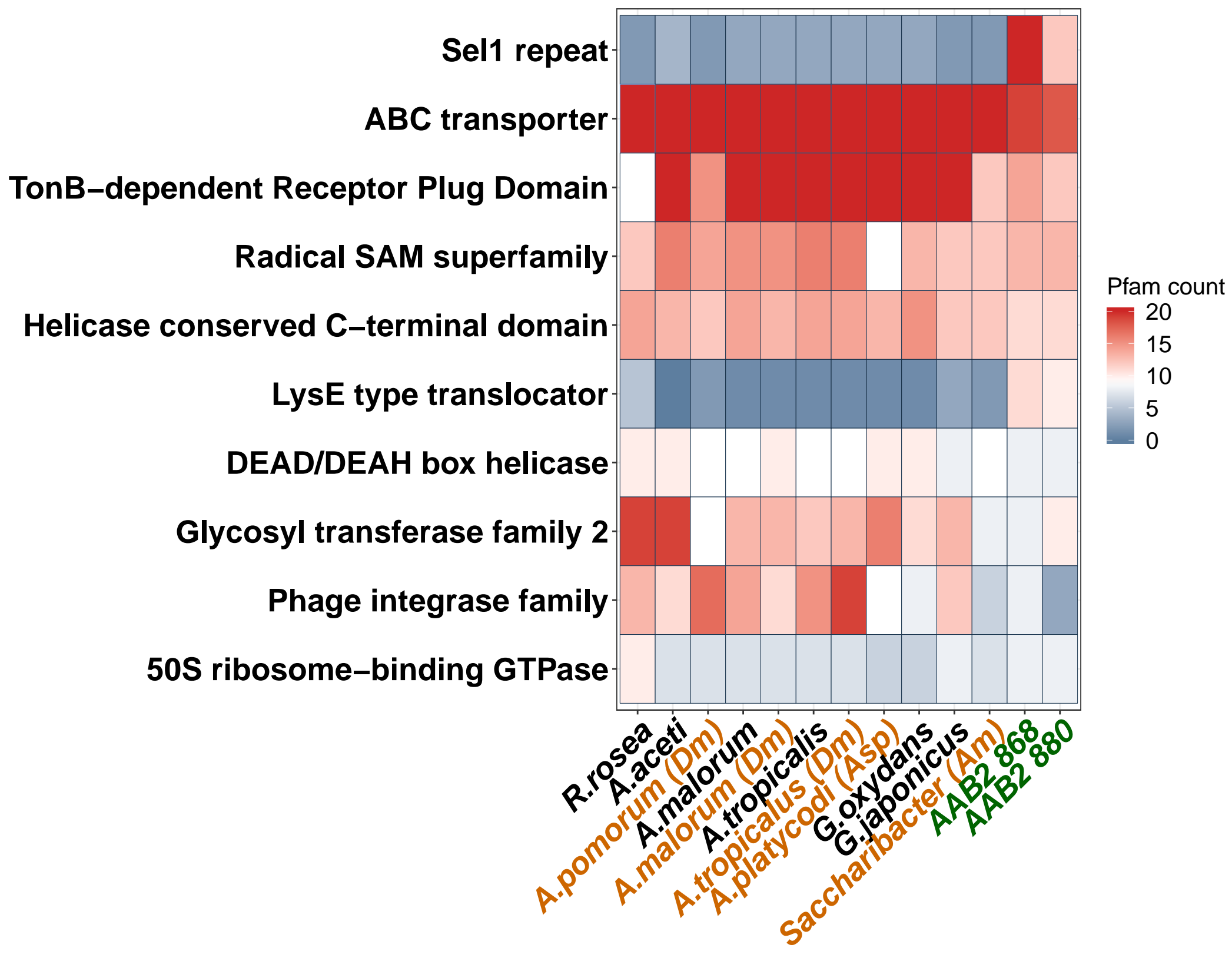

Supplement: Supplementary file 7 — Figure S7. Abundance of PFam annotations across the genomes of several free living and host associated AAB. The ten most abundant function categories within the genomes of AAB isolates as annotated by PFam category. Labels of ant associates fully sequenced in this study are colored green, other host associated bacterial isolates are colored orange, and environmental isolates are colored black. Hosts are indicated in parentheses, where applicable (Dm: Drosophila melanogaster; Asp: Anopheles species; Am: Apis mellifera). (PDF 6 kb) [file 12864_2019_5844_MOESM7_ESM.pdf]

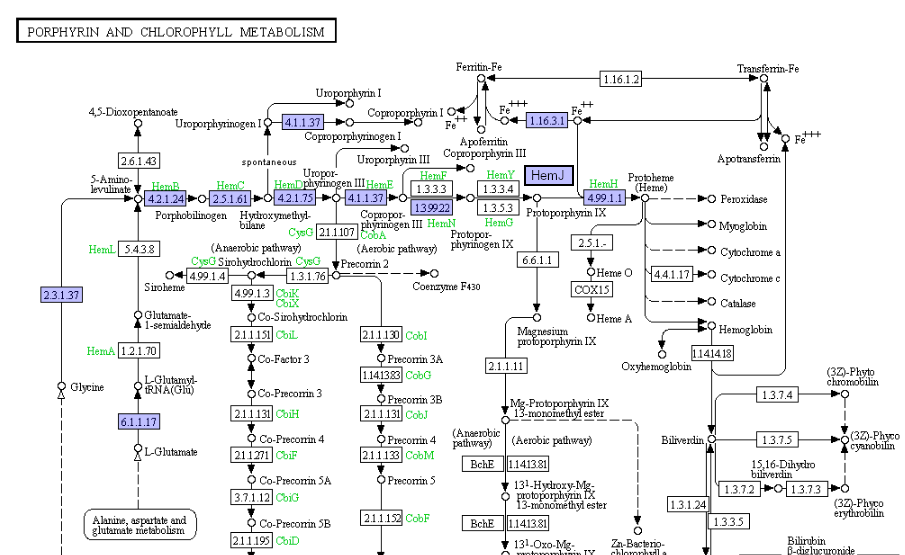

Supplement: Supplementary file 8 — Figure S8. Heme biosynthesis in AAB2 strains 868 and 880. Genes present in both strains are colored purple. (PNG 168 kb) [file 12864_2019_5844_MOESM8_ESM.png]
